# Epistemic Standards for Participatory Technology Assessment: Suggestions Based Upon Well-Ordered Science

**DOI:** 10.1007/s11948-020-00211-7

**Published:** 2020-04-01

**Authors:** Juan M. Durán, Zachary Pirtle

**Affiliations:** 1 Faculty of Technology, Policy and Management, TU Delft, Building 31 – B 4.310 - Jaffalaan 5, 2628 BX Delft, The Netherlands; 2 NASA Headquarters, 300 E Street SW, Washington, DC 20024 USA

**Keywords:** Well-Ordered Science, Participatory technology assessment, Science policy, NASA expert and citizen assessment of science and technology

## Abstract

When one wants to use citizen input to inform policy, what should the standards of informedness on the part of the citizens be? While there are moral reasons to allow every citizen to participate and have a voice on every issue, regardless of education and involvement, designers of participatory assessments have to make decisions about how to structure deliberations as well as how much background information and deliberation time to provide to participants. After assessing different frameworks for the relationship between science and society, we use Philip Kitcher's framework of Well-Ordered Science to propose an epistemic standard on how citizen deliberations should be structured. We explore what potential standards follow from this epistemic framework focusing on significance versus scientific and engineering expertise. We argue that citizens should be tutored on the historical context of why scientific questions became significant and deemed scientifically and socially valuable, and if citizens report that they are capable of weighing in on an issue then they should be able to do so. We explore what this standard can mean by looking at actual citizen deliberations tied to the 2014 NASA ECAST Asteroid Initiative Citizen forums. We code different vignettes of citizens debating alternative approaches for Mars exploration based upon what level of information seemed to be sufficient for them to feel comfortable in making a policy position. The analysis provides recommendations on how to design and assess future citizen assessments grounded in properly conveying the historical value context surrounding a scientific issue and trusting citizens to seek out sufficient information to deliberate.

## Introduction

Participatory technology assessment (pTA) has been developed as a way to “engage a representative group of lay people in the processes of science and technology decision-making” (Sclove 2012).^1^ It is in some ways an outgrowth of studies on technology’s role in society that began in response to concerns over U.S. R&D in the 1960s and which led to the establishment of the U.S. Office of Technology Assessment (OTA) in 1972. Until its disbandment in 1994 due to budget cuts, the OTA performed a series of technical analyses for Congress using unprecedented assessment techniques. While the OTA has been criticized for various reasons, it did help formalize tools and create interest that fueled a larger technology assessment (TA) community (Morgan and Peha 2003; National Academy of Public Administration 2019). A chief appeal of pTA in its different variants stems from empowering the public to consider decisions that some might otherwise think a lay public would be incapable of doing.

The broad focus of pTA is about involving citizens in the assessment of values and the informing of decisions. The problem of assessing the societal value of various research and development efforts is an old topic that continues to generate significant research [Bozeman and Sarewitz 2011; Coates et al. 2001; Guston 1994 (discussing an 1880s case); Mowery and Rosenberg 1979]. Despite all of this debate, there is no systematic governmental method for thinking through the value of research that extends above and beyond some form of financial return (Bozeman and Sarewitz 2011). pTA is one of very few prospective methods for assessing public values inside of research, and it can be a natural fit with the upstream engineering concept we allude to in this article. The strand of pTA efforts that was ignited by Sclove (2012) has been developed in the U.S. by the Expert and Citizen Assessment of Science and Technology (ECAST) group, which has engaged in a series of pTA efforts on climate resilience, geoengineering, asteroid exploration, and autonomous vehicles (ECAST 2018). This work has supported a mix of clients ranging from government agencies, private foundations, and universities. There are other varieties of participatory research, including citizen juries as well as other European approaches to pTA (Einsiedel et al. 2001; Joss and Bellucci 2002; Klüver et al. 2000).

In reviewing some of the results from these pTA citizen forums, the question is often asked whether citizens have a sufficient technical understanding of the issues to be able to discuss them in these forums. While the pTA literature has developed standards on how to evaluate such forums, especially arguing that deliberation results need to be integrated into a design system that grows through iterative feedback (Chilvers and Kearnes 2016; Dryzek 2010), there has been relatively little development of standards for what it means for a citizen to be ‘well informed’ in pTA. Practitioners who run pTA exercises often put in controls to monitor the quality of a dialog and to ask pre/post interview questions assessing the knowledge of participants. Given the virtues of public participation, it can be questioned as to whether pTA designers need ideal epistemic standards for what citizens should know and think when they are engaging in pTAs. There are other analogs where citizens are used to make decisions, such as jury trials, where it is commonly assumed that citizens’ judgments should be respected and assessed. Yet some of the literature on science policy does embrace a generally technocratic mindset, and this may overly incline people to question the involvement of citizens in scientific issues.

In this article, we seek to develop an epistemic standard that could be applied towards pTA research efforts to provide a more foundational basis for understanding what citizens should know and understand within pTA. We want to ground our standard in an established science policy model, and therefore we will first explore several models of science policy to seek a relevant basis for our proposed standard. While recognizing that other choices and epistemic standards can be developed, we favor the standard based on Philip Kitcher’s notion of Well-Ordered Science (WOS) (Kitcher 2001). Adopting WOS for our claim is key for our approach because it requires ideal deliberants to be tutored in historical understanding of the scientific significance and values surrounding a research question, and for the citizens themselves to feel ready to debate on a given science and technology issue. Experts in this standard can still be helpful in establishing how to pursue higher level goals (e.g., national research agenda, establishing new national laboratories), however, they play no central role in this model of science policy.

Taking these principles from WOS, we want to illustrate what this standard entails by applying it to a case study of a pTA effort. To that end, we discuss the 2014 NASA ECAST Asteroid Initiative pTA. We identify many features of values focused on deliberation that are similar to the WOS-derived standard that we explore. We also suggest that the forum should have provided more background context on the historical development of the significance for Mars exploration, to help meet the standard.

While it may not be needed by practitioners who already know how to implement pTA activities, our contribution here is to provide a plausible new epistemic standard for being informed in pTA. The approach we offer is a balance. By reflecting on the mix of historical values and epistemic significance in deliberations, public dialog can be enriched in a way that is different from the broader technological milieu. WOS offers means to entrench and evaluate the significance of scientific and technological research. But WOS also offers a way to introduce an epistemic standard on being tutored, pivoting away from notions of being informed that hinge on some test about overall knowledge of facts and processes of individuals. Being tutored in Kitcher’s sense is more about the awareness of other citizens’ values and about understanding the history of why certain scientific questions came to be seen as valuable. The standard we develop, then, provides normative guidance to many pTA efforts intended to only partially depend on the expert review, but which also focus on the knowledge acquired in the process of being tutored and which has historic value that makes research meaningful.

The article is structured as follows. “Five Models for Science Policy” section discusses five different models for science policy. Based on the variables *participation in decision making*, *affected group* and *general education of participants*, we show that WOS is the only model that can plausibly give useful standards to pTA efforts, such as the NASA ECAST Asteroid Initiative pTA. “Well-Ordered Science: Key Concepts” section addresses key concepts in WOS that are at the heart of our claim of epistemic standards applied in pTA efforts. Examples of these concepts are *tutoring*, *scientific significance*, and the *role of scientific and engineering experts* (henceforth *experts*) *and citizens in an ideal deliberation*. With these ideas firmly in mind, “Epistemic Standards for pTA from WOS” section addresses the main claim of this article which is that WOS can offer epistemic standards that can be applied towards pTA research efforts. “NASA ECAST pTA Overview and Coding Approach”, “Results of Background Content Assessment, Vignettes and Coding of Debates” and “Analysis” sections discuss in depth the NASA ECAST Asteroid Initiative as a case study of pTA. “NASA ECAST pTA Overview and Coding Approach” section reviews the Asteroid Initiative and discusses approaches to coding; “Results of Background Content Assessment, Vignettes and Coding of Debates” section complements the review by presenting the results of the debate; finally, “Analysis” section presents the example in terms of what standards should be required in practice. “Conclusion” section addresses final remarks regarding the potential epistemic standards from WOS for real-world pTAs. We also add an “Appendix” with background material used for the citizen forum.

## Five Models for Science Policy

In order to assess what citizen deliberations should be, we decided to choose a model framework on how science policy should operate and which might offer normative guidance for pTA. Studies in science policy offer a series of models capable of describing and accommodating different institutional policies and practices. Logar (2011) does a systematic review of five science policy models, and we are following his conclusions. We will only briefly review the science policy models here due to considerations of space.^2^ These are, the *linear model* by Bush (1945), *Mode 2* by Gibbons et al. (1994), *Post-Normal Science model* by Funtowicz and Ravetz (1993, §), *Pasteur’s quadrant* by Stokes (2011), and *Well-Ordered Science* (WOS) by Kitcher (2001) (see Logar 2011, p. 251). Although each model has its merits, we believe that WOS is the most suitable model for accommodating pTA.

Our reason for choosing WOS is that the model enables modes of participatory involvement of citizens that better reflect the kind of practice and epistemic standards for methodologies such as pTA. A main characteristic of pTA is being ‘participatory’ incorporating a broad range of the public. The forms of how participation should occur is open to discussion, as it could take different forms (e.g., surveys, meetings, etc.) and be interpreted in different ways by the models for science policy. We do recognize that other models do have participatory elements. For instance, in the *linear model*, the participants in the decision making are scientists. Similarly, in the case of *Mode 2*, participation “is achieved through involving an extended group of experts, including lay ‘experts”’ (Gibbons et al. 1994, p. 83). But these models suffer from major disadvantages. One is that they seem to be at odds with pTA. For instance, the linear model is primarily focused on the organization of scientific research, which is distinguished from its applications (of which technology is just one of them). However, we should not expect the linear model to have anything relevant to say about incorporating the public into discussions about technology. Instead, scientists (and not necessarily experts in technology) are chief participants involved in such discussions.^3^ Another major disadvantage of these models is that they could lead to an overly expert-driven and technocratic form of democratic participation through the heavy reliance on expert advisors.^4^ In this respect, the form of participation proposed by these two models is in conflict with pTA and is unlikely to be able to provide the kind of helpful epistemic standards that we are seeking here.

Unlike the *linear model* and *Mode 2*, in the *Post-Normal Science model* participation is understood as an ‘extended peer community,’ and in this respect takes it that “[w]ith mutual respect among various perspectives and forms of knowing, there is a possibility for the development of a genuine and effective democratic element in the life of science” (Funtowicz and Ravetz 1993, p. 741). Advocates of the Post-Normal Science model take that ‘extended peer community’ must be understood as “the relevant peer community [that] is extended beyond one particular research community, to include users of all sorts, and also managers” (Funtowicz and Ravetz 1993, p. 746). Indeed, to Funtowicz and Ravetz, participation is extended beyond the direct designers, producers, sponsors and users of a given research, but it also includes investigative journalists, ethicists, lawyers and groups related to the research in question (Funtowicz and Ravetz 1993, p. 752). A chief characteristic of the Post-Normal Science model, then, is that the participation of the stakeholders presupposes some substantial vested interests in the project. As Funtowicz and Ravetz explain, these interests are of the technical stakeholders and experts on the problem at hand as well as people directly affected by or involved in such problem. The example of the AIDS community is paradigmatic: doctors, nurses, journalists, patients, families of patients, researchers, pharmaceutical companies, and government agencies all having vested interests in improving the quality of life of patients. In principle, this view on the stakeholders and their role in Post-Normal Science differs from pTA efforts, where the peer community is extended to include “the public,” that is, citizens with no predetermined links to a given research and who do not necessarily have a substantial stake in the project. Thus understood, a lay-person that participates in a pTA might do so because of a number of reasons: she is aware of the health problem that AIDS poses to society at large, she is interested in eliminating the social stigma that AIDS patients have had to deal with for so many years, and so on. But in principle, this person is neither affected by the infection, nor has a close relative suffering it, and surely has no financial motivations.

This led us to reflect more deeply about choosing between the Post-Normal Science model and WOS as candidate models to use to create epistemic standards. Both include non-expert citizens as part of their model of participation in decision making, although WOS includes citizens without vested interests in the problem at hand. The crucial difference between both, and ultimately the reason for preferring WOS over the Post-Normal Science model, is that the former requires participants who are representative of all of society, rather than including those who are directly involved in the policy topic being deliberated on. Indeed, in the Post-Normal Science model, the participants are persons directly affected by the issue at hand because they have a keener awareness of the specifics of a given problem, and more pressing concerns with the solutions provided by institutions. In other words, the participants in the Post-Normal Science model are those directly affected by a specific issue, and not a citizen concerned about the general well-being of her fellow citizens, the reputation of her country’s institutions, or even the general direction that scientific and engineering research could take.

To complete our justification for focusing on WOS, it is not sufficient to rule out alternative models of policy making, but reasons for choosing WOS must also be given. The practice of science as described by WOS is appealing to our purposes for several reasons. The principal rationale for deliberation is to specify the problems to be pursued by science as they would be endorsed in a democratic deliberation, albeit in a deliberation that its designer, Kitcher, calls idealized and hypothetical. In WOS, tutored participants participate under conditions of mutual engagement, wherein each deliberant is assumed to be willing to go beyond their own prior viewpoints in ways that real world participants may resist. Further, participants are citizens of all social strata. Each with different concerns and motivated by different reasons. Some citizens may want to promote science and technological achievements, others may think that real democracy is based on their civic responsibility to get involved. Yet others engage in democratic deliberations because they recognize scientific knowledge as a human good—not for Americans or for the rich, not for intellectuals or the well-educated. Unlike the Post-Normal Science model, in WOS, citizens can be motivated for several, and quite different reasons, making it a more democratic engagement. While there are idealized aspects of deliberation, such as the willingness of participants to openly engage and the ability to have a large and structured public deliberation, many aspects of the ideal can be implemented (cf. Brown 2004; Pirtle and Tomblin 2017).

## Well-Ordered Science: Key Concepts

The WOS ideal posits a deliberation to set out the goals of science as laid out in the following table from Pirtle and Szajnfarber (2017) (Table 1).

**Table 1:** Kitcher’s Well-Ordered Science (WOS)

Assumes democracy is the best way to identify society’s goals and morals^a^
Imagine that representatives of society come together
• with tutoring necessary to understand science policy decisions
• understand and respect the preferences of the other representatives
• committed to making a decision by voting
In WOS, this group would vote and make science policy decisions at three levels^b^
1. The arbiters would decide on what the goals for scientific research should be
2. With the help of experts, the arbiters would approve of means to pursue and achieve these goals^c^
3. Once research yields results, the arbiters would decide how to spread the benefits amongst society
While Kitcher holds WOS to be infeasible in practice, he believes it holds value as a thought experiment. Scientists may reflect on their research and whether it would achieve the goals and meet the moral standards that would exist in a state of Well-Ordered Science

^a^We thank an anonymous reviewer for pointing out a tension existing within Kitcher’s own viewpoints on WOS. Indeed, Kitcher seems to waive between considering democracy as a way to identify society’s goals and “fair-share principles” (Reiss and Kitcher 2009). To this reviewer, the latter suggests that we already have moral principles not necessarily justified democratically determining some cases of funding. This tension does not affect our main thesis

^b^It is worth noting that Pirtle and Szajnfarber base their table on Kitcher’s (2001) book. However, in Kitcher (2011) the image is more complex than of a group deciding by means of some voting mechanism. In fact, the commitment to voting is Kitcher’s last resort. The best option for Kitcher is to go for what is best for everyone; the second-best option is to go for what is acceptable to everyone; and the third-best option, where “[if] voting ever occurs, it is a matter of last resort, when we reluctantly agree that consensus is impossible” (Kitcher 2011, p. 114), that is, majority voting. We thank an anonymous reviewer for clarifying this point for us

^c^This should not be understood as assuming that anyone could reliably predict the means that will attain specific goals. This is an important point that requires further discussion. Unfortunately, we cannot address them here. For discussions, see Acuna et al. (2012), Gomory (1995), Penner et al. (2013), Shaw (2018) and Stokes (2011, pp. 100–102). We thank an anonymous reviewer for bringing this to our attention

By reflecting on WOS, we want to provide epistemic guidance on potential standards for engaging in Participatory Technology Assessment (pTA). This is to say, that we must make clear the concepts involved in WOS, their involvement in a democratic ideal, and how this is later appropriated by pTA. In the following, we discuss three central concepts in WOS which are central for our understanding of pTA. The first is the idea of *scientific significance*, that is, the question of what is worth debating. Time, budget, and a plethora of other scarce resources make plain the fact that there cannot be deliberation of every single scientific or technological issue, rather it must be carefully decided how to best allocate such resources. The second important concept is for a participant to be *tutored*, which we will show is not the same as expertise. As mentioned, in a pTA forum, citizens are being tutored in such a way that they are able to make well-informed decisions. This concept is directly related to the notion of *expert* and *expertise*. As we have mentioned above, experts are a central element in almost every other science policy model, to such an extent that the process of decision making is stopped in case of their absence. One can question whether experts are an important player in WOS, and thus it is largely related to other models, or their presence is secondary to the point of being necessary in special cases. We will show that WOS treats experts as having a secondary importance when it comes to establishing the goals of scientific research. Finally, the third key concept is how WOS depends on the tutored participants engaging in an idealized democratic deliberation. We shall discuss what this means and how it might relate to standards for engaging in pTA.

### Scientific Significance

The underlying problem of scientific significance can be interpreted as the question of the aims of science.^5^ Or, if you prefer, significance questions the purpose of scientific inquiry. Many have argued that science is directed towards finding truths about the world (e.g., Proctor 1991 and Douglas 2009, Ch. 3). As is known, those truths come in different flavors: by explaining and thus obtaining some form of understanding (Friedman 1974), by discovering causal processes that bring about phenomena (Salmon 1998), by identifying the fundamental laws and principles of nature (van Fraassen 1989), or portraying a unified picture of the world (Morrison 2000). But as Kitcher (2001, 2011, 2019)—and many others have pointed out—there are many truths about this world that are not worth any form of scientific inquiry. For instance, an explanation of why an individual does not wear shorts in summer might be of interest to someone close to this person, but certainly it is not worthy of any scientific inquiry. Kitcher uses examples such as efforts to count pebbles on a beach as generally not adding to a body of significant knowledge. Of course, one could think of examples that cast some doubts that the notion of significance can be easily determined. To see why the historical context matters, consider for instance an explanation of why the sun rises in the East. Whereas obtaining such an explanation *was* scientifically significant in the past, it would be puzzling to think of it in the same way today. In this respect, Kitcher sees scientific significance as concrete, socio-economic projects for the general good (Kitcher 2001, p. 207). Likewise, the aims of the sciences—and, in some cases, of engineering as well—are not merely to attain some practical goal, and many humans are driven by curiosity and wonder.^6^ Much of so-called basic science seems to lack such a practical-goal component. What, then, are the aims of scientific inquiry? In other words, what is scientifically significant?

Examples of scientifically significant research and claims vary greatly. Some have a linguistic basis, making statements about finding new theories, generalizing statements, and predicting new states of the world. Some types of research are non-linguistic, such as seeking new entities, using new techniques, opening new lines of investigation—and perhaps creating a new school of thought. These examples align with general disciplines, such as astronomy and the significance of answering the fundamental questions about the origins of the universe; and biology, such as addressing the problem of defining what life is. But they also include more problem-focused fields, such as environmental studies, studies on cancer, and new crops in agriculture. Scientific significance spreads across disciplines, fields, and interests. Kitcher makes clear that a claim that is scientifically significant does not have to be tailored to epistemic values, nor social, nor cognitive, nor practical ones. Some issues, and this is at the heart of Kitcher’s thinking, are scientifically significant for their own sake, that is, because they answer human curiosity.^7^ Judgments of significance involve a multidimensional balancing act across human values, including curiosity as well as more social values. The answer of the right balance of values to pursue is decided within the WOS deliberation construct.

Scientific significance accrues to those problems that would be singled out under conditions of a WOS-deliberation and, in that respect, they are both inputs to and outputs of WOS. Kitcher elaborates on scientific significance in the case of Dolly, the cloned sheep. In this respect, it is important to note that the notion of scientific significance is context dependent and value-laden, as they are intrinsic to the practice of science. That is to say, whereas the maturation of scientific significance involves channeling science toward seemingly epistemic aims (e.g., identifying laws of nature, providing understanding of phenomena, building new theories), this must not be understood as objective and value-free. Moral and social values are part and parcel of the practice of science.^8^ As mentioned, the case of Dolly nicely illustrates the connection between scientific significance and value-laden scientific practice. On the one hand, the study of Dolly does not single out general laws of ovine development,^9^ yet it is central to scientific research to approach answers to fundamental issues, such as the possibilities of improving livestock—and therefore finding possible solutions to food shortage—and understanding cellular differentiation (Kitcher 2001, p. 76).^10^ On the other hand, as acknowledged earlier, there are the values of knowledge and scientific inquiry for their own sake. But Kitcher takes the issue further, arguing that the success of the scientific enterprise also depends on whether it advances our efforts to attain the ends that matter to humans (Kitcher 2001, p. 30).^11^

### Tutoring and the Ideal Deliberation

Tutoring, according to Kitcher, is part of a process entailed by an ideal deliberation.^12^ That is to say, members of a society willingly engage in a debate with peers under conditions of mutual respect and free discussion. To this end, participants recognize themselves and others as having different sets of ideas and desires, and that neither member is to be expected to sacrifice their own viewpoint and wishes to the preferences of others (Kitcher 2001, p. 119). Kitcher takes that the deliberation best operates under the premise that participants leave behind their prior biases and goals. Furthermore, for a full democratic debate different social, ethnic, religious, and intellectual groups must be represented, although this cannot always be achieved.

In order to prepare citizens to take part in a deliberation, Kitcher recommends the use of tutoring to inform the citizens. However, this is not about tutoring to teach scientific details and expertise. Kitcher talks about conveying a background on the historical significance of why a research problem came to be seen as important. On pages 79–80, and 118 of Kitcher (2001), he uses plots of significance graphs to illustrate how questions of cloning came to be seen as significant. The mapping out of significance graphs and understanding why an issue came to be important does require involvement from experts to inform the citizens. But the main value of tutoring here seems to be to give a map to citizens about how the values that they hold may become relevant to a given policy debate, so that they can better appreciate the nuances of a policy question being debated under WOS. Having a sense of the historical significance on why a question comes to be important is central to WOS.

Thus far we have mentioned a few characteristics of an ideal deliberation, where members of a society engage in debates under conditions of mutual respect. The process of tutoring, then, begins with each participant becoming aware of the potential scientific significance of a line of inquiry. Thus understood, tutoring can take different modes and formats, and thus they have different timeframes. Our discussion of the NASA ECAST pTA in “NASA ECAST pTA Overview and Coding Approach” section is a good example of how tutoring can be carried out. In this particular case, there is a prior process of educating the participants with basic information regarding the question at hand. Naturally, this process of educating is not meant to replace an ideal deliberation, nor participants can make informed decisions with the basic information, but rather that participants are better prepared for engaging in an ideal deliberation (e.g., by all agreeing on the same conceptual grounds).

The aim of an ideal deliberation, then, is for participants to become aware of the potential significance—epistemic, practical, social—of possible lines of scientific and technological inquiry. Furthermore, participants must be able to explain to other participants their own positions, as well as give reasons for opposing other participant’s viewpoints. Of course, in some ways a given scientific significance can change form as you get into the deliberative process. People can change paths, raise new questions. As Kitcher puts it: “Personal preferences have given way to tutored personal preferences” (Kitcher 2001, p. 118). The end of an ideal deliberation is when each participant is able to accommodate their own preferences to the new information framework. That is, when they are able to evaluate how, and which possible lines of scientific and technological inquiry discussed during the deliberation are now best represented. Equally important to being tutored is being able to communicate your personal preferences to others. To resolve which course of action to take, Kitcher suggests a voting system as the most democratic way of factoring in the final decision all the participant’s preferences. This is carried out by simply first listing each participant’s preferences for a given scientific inquiry to promote (coupled with an index of intensity of interest). Then, a standard process of voting takes place to select the best option. The voting process is well described by Kitcher (2001, p. 121), including cases where no consensus is reached.

### The Place of Experts in WOS

Unlike other models of science policy, Kitcher does not grant much prominence to experts and, consequently, they do not play an absolute and defining role. In fact, experts have a rather complementary place in WOS. They are in concrete need when it is important to assess whether a particular scientific venture delivers what the ideal deliberators collectively want (Kitcher 2001, p. 119). Kitcher puts this idea in the following form: “the ideal deliberators can pick out a group of people to whom they defer on scientific matters generally, that this group defers to a particular subgroup with respect to questions in a particular field, that that subgroup defers to a particular sub-subgroup with respect to questions in a particular subfield, and so forth” (Kitcher 2001, p. 120).

In this respect, experts are different from ideal deliberators.^13^ Particularly, in their influence on questions of scientific significance, their capacity to make informed decisions, and their impact on scientific research. In plain words, experts see their epistemic authority circumscribed while some of the decision-making power is given back to concerned citizens. Kitcher thus inverts the standard relation of the expert presence superseding participants and sets experts as being referred to by participants when in need. This, we believe, is the ultimate aim of science in a democratic society.^14^

## Epistemic Standards for pTA from WOS

A chief aim of this article is to understand Participatory Technology Assessment in the context of Well-Ordered Science. To this end, we must understand how pTA relates to WOS.

Let us begin with questions of scientific significance. The sources from which researchers and the public consider a question to be scientifically significant can be various and diverse. There can be unstated values in past research that are motivating a scientific question that, in the right context, the public would identify and be able to assess the value of today (Elliot and McKaughan 2009). By carrying out a pTA, one can obtain a richer, potentially broader (or even different) sense of the values underlying scientific and technological research. For instance, one could attain very different perspectives on the value of research on genetic differences between men and women, or issues related to human consciousness. Chronologically speaking, questions of scientific significance are not a priori, determined prior to carrying out a pTA, but rather they are actively assessed and determined through the pTA. There are, nevertheless, judgments and perceptions of scientific significance that have been around for some time, that have been known and assessed prior (and perhaps are the reason for) carrying out a pTA. Take for instance research on cloning and the fundamental research questions about how cells behave and what composes an organism. An initial sense of significance can be examined beforehand and then described and used as background material in a pTA forum.^15^

Besides having the right question, a crucial element in any pTA is to determine when citizens are tutored and able to deliberate. Unfortunately, Kitcher does not provide clear criteria that enables us to establish when citizens are tutored and thus when they can make informed decisions. Let us see if we can make clear what the context of being tutored in a pTA exercise might look like, based on an inference from the logic of WOS.

The processing of tutoring in pTA begins with the background material provided during the first states in the forum. Ideally, such material would cover the relevant facts and data about selected questions on scientific significance, which Kitcher implies has a sense of why a scientific question came to become important. Now, such material would as a result be scientifically and technologically valuable, as well as provide a historical context about the social value of a given research question. Defining the process of tutoring in this way means that pTA participants do not need to be assessed based on their expertise, previous knowledge, or being directly affected by the issue addressed in the pTA. Rather, it reaches out to citizens with different concerns and different aims, directly assessing issues of public value. WOS is, in this sense, a relevant and appropriate normative and theoretical framework for practicing pTA.^16^

The process of a pTA deliberation is carried out in a similar fashion as Kitcher describes an ideal deliberation. Participants are expected to engage in debates with their peers under conditions of mutual respect and free discussion.^17^ Norms of cooperation are identified prior to a deliberation, and table facilitators help to ensure that no one dominates the conversation. Participants can be tutored in a pTA context, when they are ready to explain to other participants their own goals and desires about the core topic of the pTA. Of course, in the real world some individuals can be intransigent, and unwilling to cooperate or broaden their worldviews. There is a sense in which the WOS-assumption of perfect deliberators is impossible to achieve in the world, but in many public deliberations the willingness of participants to behave in a way that is close to the ideal may be high, and close enough to mimic the deliberative benefits of the ideal WOS concept.

Given the above discussion, there can appear to be a few high-level metrics about whether WOS-like conditions are being applied. If there is no discussion of historical context on the values of key research questions, so as to allow assessment of scientific significance, then there is a relatively clear gap. But it can be difficult to reflect on what level of detail to include on relevant scientific and technical information and description of historical significance on values is appropriate. To be tutored in a pTA context is not an instance of an objectively quantifiable educational goal, but rather tutoring is anchored in the first-person viewpoint. Thus, we take it as cases where participants feel they are ready to deliberate and make opinions about an issue and they inform others of their individual views without claims of states of fact. This raises the question of, when can one establish that a given participant has been tutored? Based on the internal logic of WOS,^18^ we submit that participants are tutored when they achieve certain goals as expounded by the organizers of the pTA (e.g., to understand the advantages and disadvantages explained in the material given during the pTA, to be able to assess and evaluate their own opinions for further discussions with others, to expose their own opinions in a clear, orderly manner, and similar activities). The key is that someone feels that they have the ability to deliberate on something; if a participant feels so, then their knowledge is good enough and we can treat them as tutored. As shown in “NASA ECAST pTA Overview and Coding Approach” section, these points can only be determined on a case by case basis, as it depends on institutional goals, preparation of the pTA (i.e., its methodology, propaedeutics, etc.), the participatory citizenship, and the like.

Now, a standard process of deliberation in pTA might include at the end, some significant involvement of experts. Although worrying about levels of expertise is not key to Kitcher’s framework, it is important to acknowledge that experts are present in pTA.^19^ In fact, experts also help (but not exclusively so) in determining what background information on scientific significance is presented, which strongly shapes how to decide if a participant has been tutored. pTA practices, then, acknowledge the role and importance of experts without subscribing to a technocratic practice where experts decide on the outcome of a pTA.

## NASA ECAST pTA Overview and Coding Approach

Kitcher’s WOS and notion of tutoring offers an epistemic standard that can be applied to real-world pTA efforts. The specific guidance for pTA would be to have a plausibly sufficient amount of context on public values so that scientific significance can be assessed and then, based on that, the deliberation can be seen as having value if the deliberators feel that they are capable of weighing in on the problem. In order to assess how the theory of WOS might apply to a real-life case of pTA, we will discuss a relatively recent example to explore what a notion of tutoring would look like in a public deliberation. The goal is not to provide a normative stance or position based on this one case study, as that would be an overgeneralization. Rather, we want to use a real-world case to explore what it would look like for Kitcher’s notion of epistemic significance and the first-person self-determination of tutoring to play out. While we can contrast and see ways in which this case study embodies a WOS-like process, the goal is to provide a more detailed empirical framing of what a standard for tutoring should look like.

### Case Study

In 2014, NASA and the Expert and Citizen Assessment of Science and Technology (ECAST) network cooperatively agreed to implement a pTA on NASA’s Asteroid Initiative.^20^ This initiative was a combination of NASA’s work to inform planetary defense, such as detecting asteroid threats and preventing them, as well as to explore asteroids with human beings as part of the agency’s overall Mars mission plans. This was done as an effort to deliberately get citizen input prior to making a major technical decision. The results of this citizen deliberation have been discussed in Bertrand et al. (2017), Pirtle and Tomblin (2017) and Tomblin et al. (2015a, b, 2017).

We will briefly review how this forum came to be and the pertinent details of this pTA case. The ECAST partners were responsible for developing objective content based on input provided by NASA. They solicited NASA managers about issues and questions that the agency wanted to get public input on. ECAST then implemented the forum by soliciting several hundred applicants for 183 in person deliberation slots. ECAST selected candidates to achieve a representative participant set that matched the demographics of the state where the forums were being held, which in this case included Arizona and Boston (MA). On the day of, citizens were divided into groups of six to eight people, guided by a facilitator. The participants had a set of background material that they had received in advance (see Tomblin et al. 2015b) and which was then shown to the entire audience through a clarifying video presentation that provided images to match the content. Participants could propose new ideas that went beyond options provided by ECAST.

The Mars deliberation at the end of the forum day assessed different options for how NASA could explore Mars (see Bertrand et al. 2017 for more details). The question supposed that NASA had three options for exploring Mars after it had established its initial capabilities: a joint orbital and robotic approach with crew in Mars orbit and robotic probes on the ground; a brief human voyage to Mars surface; and a push for permanent human settlement of Mars surface. Participants were provided context about the challenges of developing Mars missions and were informed about the cost and schedule tradeoffs associated with pursuing more rigorous missions to Mars.

Data was collected from the citizen participants in several ways, with some tables receiving recorders and transcriptions, having in person observers, with participants completing pre-/post-surveys, and with participants writing down their opinions and rationales both as individuals as well as their collective group opinion (although they did not write a group rationale).

### Method for Assessing Case Study

While the forum was primarily focused on issues tied to asteroids, our content analysis here will focus on an hour-long deliberation at the end of the day on different strategies for human exploration of Mars, which at the time was the planned follow-on to human exploration of asteroids. This Mars deliberation is a representative portion of the overall NASA ECAST forum, and we chose to focus on it because prior research had created tabletop transcripts and a combination of detailed written responses to allow us to properly explore the case. We also assess the background material to explore to what extent it provided an overview of epistemic significance as laid out by Kitcher.

This coding analysis focuses on every transcript discussion from the Mars deliberation section from the forum, which debated different mission profiles and strategies for going to Mars. For the data below we assessed seven transcripts from the table deliberations. This captured a broad, random sampling of the total number of 27 (TBR) tables across the two sites, and as a result included over 40 people in dialog. The number of transcripts taken was due to practical constraints (number of recorders on hand).

In terms of coding approach, we used an iterative, open-category approach, reviewing the data to identify relevant patterns (Bertrand et al. 2017; Glaser and Strauss 1971). As a unit of analysis, we coded every instance at which participants seemed to be debating or actively assessing a topic. This debate (or assessment) claim is captured in Table 2 below. Much of the conversations were focused on understanding and reviewing what was said in the background material, which we did not code as a debate or assessment event. Once we had the full set of 46 debates that occurred across eight tables, we assessed the dialog to see how participants approached the deliberation. We coded whether they made any references to expertise in the discussion, either about their own expertise or the need for experts to weigh in on the problem. We assessed if the discussants were able to get closure on the debate topic, and if there was a need for more information in order to resolve the topic. As a ‘heuristic’ in terms of categorizing and assessing the results, we coded whether the debate was over a matter of facts, of values (which can be deemed as more closely tied to Kitcher’s notion of scientific significance), or a mix of facts and values [see for instance Longino (1990), Magnani and Nersessian (2002) and van den Hoven et al. (2015)]. While many scholars have shown that it can be difficult to have an absolute separation between facts and value,^21^ this distinction was chosen as a heuristic to tease out if the key to closing a debate (or preventing it from being closed) was due to issues of expertise or issues of significance, as it ties to Kitcher’s notion of tutoring described above.

While we are using this case study to visualize what Kitcher’s epistemic standard of tutoring can look like, we must clarify we are not attempting to assess if this case itself represents a moral exemplar that other pTAs should follow. One can nevertheless assess real-world pTA data to see how WOS might apply to the discussion. If something like Kitcher’s notion of tutoring about significance was manifested in a real-world pTA, we would expect many if not most of the debates that occurred in the forum to hinge upon matters of value, or at least about a mix of fact and values.

In addition to coding the deliberation material, we will also assess the background material (included in the “Appendix”) for the extent to which the deliberation sufficiently discussed the historical context of the development of significance for Mars exploration. We also code the limited number of questions from the citizen deliberants that were sent via an online program to get answers from NASA experts, coding them along the same axis of fact and values discussion shown above.

## Results of Background Content Assessment, Vignettes and Coding of Debates

In our overview of the background material for the citizen forum (copied in the “Appendix”), the material alludes to some public knowledge of the value context and significance of Mars exploration. However, the forum could have used a much more explicit historical overview about the reasons for human exploration. While the forum provided background material on the challenges of going to Mars and a summary of what humans would do there, a history of mission planning and why Mars has become a policy-focus for exploration was not made. Bertrand et al. (2017, p. 51) noted that the deliberants did talk actively about why Mars mattered, and made claims about its significance, but that more open-ended discussion could have been facilitated by more explicit background material. The background knowledge of values surrounding Mars and the significance of exploring it are sufficiently present in the deliberation discussions (partially evidenced by the below vignettes), and deliberants were not actively asking and demanding more information about the potential value of exploring Mars. Given this discussion, it appears that the lack of a historical background on Mars deliberation in the forum does not invalidate our using this discussion to illustrate what Kitcher’s notion of tutoring may look like in public deliberations.

Table 2 below shows a list of all debates that occurred during the Mars deliberation with the coding analysis applied. To help illustrate debates about significance and a group feeling willing to have closure on a given debate, we will go into specific vignettes of debates where participants came to an agreement or disagreed about a topic. These help to illustrate the concepts of significance and how a WOS-like standard for debate might play out in a real-world pTA. Speakers are shown as Woman 1 (W1), Man 1 (M1), etc., along with facilitator (F). Table 2 then codes these conversations based on whether there was a decision reached among the deliberants, and whether that decision was based on a full consensus of those at the table.

### Vignette B.04.4: Funding of the Mission

- M3: Because government funding is wasteful, that's personally what I feel.
- W4: Yeah, I don't know how. I'm pretty hesitant about the cost of the colony or of sending people in general. But, relying on private enterprises… it's like that different motivation problem again: what science would we actually be accomplishing then?
- F: Yeah, it would no longer be a public good that we can all enjoy together. It would be owned by someone.
- W3: To me it seems like they've already calculated how much money it is going to cost, and, apparently it was so much that they're so ashamed to even tell you how much it's going to be.

#### Analysis

There was no decision or closure implied in the discussion about the value of public funding, though values-focused comments were made about potential scientific output, enjoyment of Mars and opportunity costs. This is an instance where additional facts about the expected costs of a Mars mission could have influenced the discussion outcome, as the lack of information seemed to increase uncertainty and raised suspicion. There is not a deep historical dialog about the historical context of why Mars exploration became interesting, which might have helped. The standard of tutoring we derive from Kitcher might suggest that the historical context be more clearly examined in the background material, and also that if the public wants more information on costs, that it should be provided. There can be potentially large uncertainties, however, tied to architecture assessments and costs (Keller et al. 2014), so there could be challenges in knowing what accurate information to provide.

### Vignette B12.7-8: Pioneer Strategy

- F: What do you make of the Earth independent living?
- W1: That we should take care of the Earth so we don't need to live in fear.
- W2: That might be a better option.
- M3: But again, it gives you the opportunity to get out of dodge.
- W2: So are we just going to use up resources and then go exploit other planets?
- M3: Ever see the moving Wall-e? There you go.
- M2: Yeah, but that just opens up another whole door of the great minority that have a lot of money and don't care about anybody else.
- W3: That's true.
- M2: They'll just buy their ticket up.

#### Analysis

This vignette shows people discussing different rationales for going into space, with different people each teasing out alternative values on why living away from Earth is good. It raises questions of economic exploitation and environmental stability on Earth, which are questions of how existing societal values will become manifest in future exploration, as well as the predictive likelihood that something like this would occur. This is coded as a mix of fact and values here, and there is no decision or consensus that emerged from the discussion. In terms of applying Kitcher’s notion of tutoring and the extent to which expertise versus knowledge of historical context is driving this deliberation, the results are mixed. There was not a dedicated overview on the historical context of Mars exploration provided in the background material, nor a discussion of the significance of exploration. This central discussion seems to be predictions about how socio-technical and economic systems will emerge in inter-planetary discussions. This is a potential matter of sociological prediction (which could arguably be expertise driven), but also raises the more normative question of what sort of world the deliberants might want to create.

### Vignette B.04.3: Risk to Human Life

- M1: If the astronauts themselves are willing to sign a waiver saying, I understand the risk, my family won't sue anyone after I'm dead, then I think it's totally fine. At the end of the day it's their life and if they want to risk it then all the power to them.
- W1: That makes sense.
- W2: I agree.

#### Analysis

This is an example vignette where all members of a table seemed to agree on a given decision, including a value statement, which led to a group feeling more accepting to risks that astronauts might accept by landing on the Martian surface. This explicitly seems to be about recognizing various values and commitments that play into the broader decision that is made. In terms of Kitcher’s notion of tutoring, perhaps there could be additional context provided about safety tradeoffs, and also further elaboration on the questions regarding what level of safety is acceptable.

### Vignette P.3.4: Robotic Option

- F: Are robots compelling and exciting enough?
- M2: No.
- W3: Not everything has to be exciting.
- W1: It is kind of what we're saying. It would be much more cool [*sic*] to see a human go to Mars than a robot.
- W2: But you also made the point that we need the robot to get the initial information.
- W1: That is a lot safer too.

#### Analysis

This vignette is largely focused on contrasting different values (cost, safety, excitement) along with predictive questions about the potential capability of using robots. In terms of applying Kitcher’s notion of tutoring and the extent to which expertise versus knowledge of historical context is driving this deliberation, the focus is more on discussing what should be significant. There is not a consensus that is reached on the result. The decision hinges not just on values but also on uncertainties about the effectiveness of robots getting information and the safety of sending humans to space. No deeply settled position is made by the deliberants.

Summary of Table 2: the below table notes each instance of a debate that occurred across the different tables. It lists whether it was a value- or fact-based deliberation, and if there was a collective decision that follows from the results. If a debate led to a final decision where most parties seemed to have established views, that is noted, as is whether the group decision was made with consensus.

**Table 2:** Debates during the 2014 ECAST NASA Citizen Forums (ECAST 2018)

Topic	Key uncertainty fact versus value based	Decision result?	Consensus?
Funding of the mission	Values (government role) and facts (didn't disclose the cost)	No	No
People on Mars	Values	No	No
Risk to human life	Values	Yes	No
Risk to human life	Values	No	No
Viking strategy	Values	No	Yes
Pioneer strategy	Values	No	No
Pioneer strategy	Values	No	No
Proving ground approach	Values	Yes	Yes
Strategy choices	Values	No	No
Environmental concerns	Values	No	No
Robotic option	Values	No	No
Pioneer strategy	Values	No	No
Human readiness for Mars	Values	No	No
Robotic strategy	Values	No	No
Risk to human life	Value of human life	Yes	Yes
Pioneer strategy	Values	No	No
Pioneer strategy	Facts and values	No	No
Strategy choices	Facts and values	No	No
Public interest with proving ground strategy	Facts and values	No	No
Value of human life	Facts and values	No	No
Funding of the mission	Facts and values	No	No
Proving ground approach	Facts and values	No	No
Risk to human life for pioneer strategy	Facts and values	No	No
Pioneer strategy	Facts and values	No	No
Strategy priorities	Facts and values	No	No
Proving ground/robotic	Facts and values	No	No
Proving ground	Facts and values	No	Yes
Risk to human life for pioneer strategy	Facts	No	No
Proving ground approach	Facts	Yes	Yes
People versus robots, safety	Facts	No	No
Pioneer strategy	Facts	No	No
Pioneer strategy	Facts	No	No
Proving ground approach	Facts	No	No
Proving ground approach	Facts	No	No
Robotic option/proving ground approach	Facts	No	No
Viking strategy	Facts	No	No
Proving ground approach	Facts	No	No
Proving ground approach	Facts	No	No
Strategy choices	Facts	No	No
Sci-fi versus reality	Facts	No	No
Pioneer strategy	Facts	No	No
Proving ground/robotic	Facts	No	No
Pioneer strategy	Facts	No	No
Robotic option	Facts	No	No
Mission logistics (all strategies)	Facts	No	No

## Analysis

In terms of assessing the forum based upon an epistemic standard derived from WOS sketched out above, discussion of the case study helps illustrate what that standard should require in practice. There needs to be robust discussion of values as they affect major technical decisions, and that is seen here. In squarely assessing the forum, one major issue does lie with the background material, included in the “Appendix”. It does not focus significantly on developing a background historical context on values and epistemic significance. Given that much of the citizens’ discussion still hinged on values is perhaps a testament that citizens drew on their background knowledge to piece together the rationale for why activities in space are desirable or not. The literature on the NASA ECAST effort has described the effort as a pilot and did call for more historical background to be presented.

Given our interest in applying an epistemic standard toward the citizen’s themselves, perhaps the more important question is about how the epistemic standard can apply towards assessing citizens’ deliberations. Tutoring involves more than just knowledge of details, it should also include the historical context of how an issue came to be significant and why. The epistemic standard discussed above requires a deliberating group to have a context of historical knowledge about values and scientific significance, and then feel sufficiently capable to make a decision as a group. This can be seen in the pTA case study.^22^ In looking across the debates held at the forums, many had a broad mix of values and facts in the decision process. The coding of decisions as being based on facts/values/facts and values showed that there was a broad mix of all three types of statements at the center of debate. Much of the debates that citizens took part in were focused on values, and not on factual questions. Expertise claims in the background material did not seem to dominate the overall debate.^23^

**Fig. 1:**
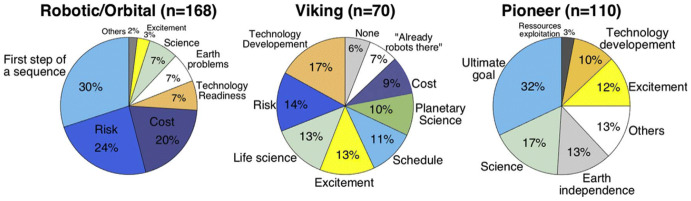
Survey of rationales used to support the different exploration scenarios

One potential path forward for better summarizing the historical context on Mars exploration’s value significance can build on some of the data derived from this pTA exercise. Bertrand and colleagues’ coding of values used to rationalize different Mars exploration approaches can serve as an initial significance map of values that the public holds to be relevant. Figure 1 above does help explain key influences that citizens referred to when they rationalized different exploration profiles. To boil these down, a smaller subset of key values can be seen as being associated with Mars exploration: (1) Curiosity (Science, Biology); (2) Value of Life (Risk, Earth independence); (3) Improved Quality of Life (Tech Development, Earth Problems); (4) Excitement; (5) Return on Investment from Public Funds (Cost, Schedule, Steps in a Sequence/Goals).^24^ While this might capture a current snapshot of values about Mars, additional work can be done to tie in historical analysis on how these values emerged and took hold. Space historians show a long-standing public interest in Mars exploration, which has been shaped and cultivated by multiple influences (Launius 2006; MacDonald 2017; Portree 2001). Other coding approaches of historical writings about space could paint a rich view of the value of Mars, which could be useful for future pTA exercises.

In terms of a group seeming prepared to weigh in on an empirical matter, we did code four cases where a participant table got to closure in debates, and also assessed what seemed to be preventing closure. Additional factual or expert-based information was generally not the key to coming to closure on a specific topic. Also, when the participants would change their minds, it was not always a result of getting new facts, as sometimes simply talking through a topic and reflecting on values seemed to lead participants to feel comfortable in agreeing on a topic (such as with the Vignette on Risk to Human Life). Participants deliberated and shared facts and values amongst themselves but were not always seeking new expertise and information. The transcripts do not seem to show a craving for more experts to inform the deliberation, although many recognized that the information they had been given received input from NASA experts.

While the coding process of characterizing debates as being tied to facts, values, or a mix of facts and values is a crude heuristic, looking at the distribution of results here is instructive for getting a feeling of how real-world pTAs may relate to a WOS deliberation. Over half of the debates were focused on values, and many decisions were based on values, separately from facts. The existence of debates on values is obvious to any scholar of policy or deliberation, but it helps establish that what a WOS-like debate would include is a lot of values debates. We highlighted above how Kitcher’s notion of epistemic significance ties into value judgments as they are shaped and affected by technical issues. An ideal WOS debate would have diverse people debating what the right values should be.

Another important result is that in no cases did the citizen participants complain that they felt they lacked sufficient expertise or information to ask a question. They did take advantage of a limited time period to ask questions, but did not return to answers to Q&A topics in conversation.^25^ While there were often disagreements among the participants, there were not major requests for new information, or those claiming that more deliberation or research would be needed. In assessing transcripts from public deliberations, there does not seem to be inherent calls for such things from the participants. In seeing how they handled value laden trades, there is in initial appearance that they trusted their views and perspectives on their own value judgments.

In assessing the data, a few other minor points seem clear. There were fewer instances of consensus among participants than expected, with only five sets of decisions reaching consensus. This may be a result of this deliberation theme not requiring consensus. Also, in considering Kitcher’s goal for citizens to be tutored, there did not appear to be major instances where desiring the historical significance was critical, or that it led to major influences. This analysis focused on a one-hour deliberation, and the historical background was underplayed in the background material, as discussed above, so this may be expected. A richer pTA discussion may want to achieve a better standard of having debates hinge on historical understanding of values and epistemic significance.

## Conclusion

A WOS-like epistemic standard for citizens deliberating in a pTA effort was developed. We discussed the standard by assessing an empirical case study of pTA as a way to help illustrate some of the nuances surrounding what an ideal versus real world deliberation should look like, where a focus on values as a proxy for significance seems essential. This data is not meant to provide a definitive aspect of how/what citizens should focus on. We are reviewing a pTA study that was designed for a separate purpose, which was to understand the public’s deliberated views on Mars exploration and other topics. There are limits to adapting such a study toward the question of determining what an ideal debate should be.

With that said, the nature of what potential epistemic standards from WOS might look like for real-world deliberants in pTA does become clearer.^26^ In reviewing citizens’ responses and assessing what they viewed their own capability to deliberate and provide advice on a topic was, the topics that they were debating heavily involved value questions in a way that a more technocratic notion of decision making might ignore. We think this aligns well with WOS’s focus on practical and epistemic significance through tutoring. Indeed, it is clear that WOS implies that an understanding of epistemic significance needs to provide some rationale on the value dimension of a question, including why it is important and how it came to be important. The standard is not about factual informedness, or the amount of facts that a citizen has at hand: such a status would be more open to challenge by outside experts.

To consumers of pTA results that have a more technocratic bent, this paper may be an important contribution in tracing how an appropriate and thought out epistemic standard for citizens’ knowledge that traces to a fundamental model of how science policy should function. Practitioners of pTA may feel the development of a standard such as this is unnecessary, to which we are highly sympathetic.^27^ Regardless, there are still practical contributions from our proposed epistemic standard that can help pTA practitioners. The standard implies that the pTA example under discussion could have done a better job in teasing out the historical dimensions of why the assessed questions about Mars exploration came to be important. To do justice to these historical issues would likely require more than just a one-day forum. The standard also affirms the value of trusting citizens who feel they are qualified to opine.

## Appendix: Journey to Mars Deliberation Background

The following are questions asked of participants during the forum deliberation.

- Which mission profile is a better choice for the future of Mars exploration.
  - Robotic exploration approach: crewed mission to Mars orbit or moons.
  - Viking approach: small-scale missions to the surface of Mars.
  - Pioneer approach: permanent human settlement on the Surface of Mars.
- What were the primary reasons for your choice?
- Do you support moving forward with the Proving Ground Strategy?
  - Not at all.
  - Not quite sure.
  - Yes, entirely.
- What were the primary reasons for your choice?
- Rank the following options for acceptability: 1 means it is acceptable, 5 means it is unacceptable.
  - A crewed mission is launched without fully qualified life support systems, since this will allow for an earlier launch date to Mars.
  - Injuries occur during the broad campaign of Mars exploration.
  - Loss of life occurs during the broad campaign of Mars exploration.
- Potential planning priorities. Rank the following options on how much of a priority you see them as. Rank 1 as “Not a priority,” and 7 as highest priority.
  - In the next 10 years, how important is it for humans to travel beyond the International Space Station?
  - In the next 50 years, how important is it for humans to travel beyond the International Space Station?
  - In the next 10 years, how important is it for a human-crewed mission to orbit Mars or one of Mars’ moons?
  - In the next 50 years, how important is it for a human-crewed mission to orbit Mars or one of Mars’ moons?
  - In the next 10 years, how important is it for humans to step foot on Mars?
  - In the next 50 years, how important is it for humans to step foot on Mars?
  - In the next 10 years, how important is it for humans to establish a permanent presence on Mars?
  - In the next 50 years, how important is it for humans to establish a permanent presence on Mars?

### Background Material, Excerpted from Tomblin et al. (2015b)

#### Session Four Journey to Mars: Planning the Future of Human Space Exploration

The complexities and challenges that need to be faced when planning a crewed mission to the planet Mars are vast. The incredible distances and harsh conditions render such an endeavor many times more difficult than any previous space initiative. While developing a plan for what a Mars mission would look like is important, it is also of critical importance to look towards the more immediate future and decide what agencies like NASA can be doing in preparation for the most ambitious exploration agenda in human history. What are the problems that engineers, and astronauts must solve when planning for Mars, and what framework will result in the most rapid, cost-effective, and successful navigating of those challenges?

*What considerations need to be addressed when planning a mission to Mars?*

- *Timeframes* While the Earth and Moon vary in distance very little over time, the position of Mars relative to Earth can change quite drastically on the timescale of months. This limits the windows of opportunity for launching spacecraft and also constrains the time it takes to reach Mars. A typical travel time for the spacecraft that have been launched to Mars is 5 months; compare this to 3 days for astronauts to reach the Moon. Advanced propulsion technologies could cut this down significantly.
- *Radiation* Mars lacks a magnetic field. On the Earth, this shields us from much of the Sun’s harmful radiation. While traveling to Mars for many months and once at the red planet, astronauts can expect to receive a significant amount of radiation. Spacecraft and habitats that are capable of protecting the astronauts will need to be developed, but even with such technology, the risk of negative health effects remains significant.
- *Surface conditions* The atmosphere of Mars is two hundred times thinner than that of the Earth and composed primarily of carbon dioxide. The surface temperatures can drop as far as − 200° Fahrenheit. Advanced habitats and space suits would need to be designed in order to maintain survivable conditions on the surface of Mars for many months at a time.
- *Supplies and fuel* Due to the distances involved, resupplying a Mars mission from Earth is impractical unless planned out months in advance. If critical habitat components fail, relying on backup parts to arrive from Earth may not be possible. With the advance of technologies such as 3D printing and in-situ resource utilization, astronauts may be able to manufacture their own fuel, air, water, and other supplies from resources found on Mars.
- *Phobos and Deimos* Mars has two small moons which are thought to be captures asteroids. While the Earth’s moon is over 3000 km in diameter, neither Phobos nor Deimos is larger than 27 km in any dimension. Even though the distance required to travel to these moons is the same as traveling to Mars, their much lower mass results in a far easier landing/takeoff procedure. For this reason, a crewed mission to Phobos or Deimos would necessitate a lower level of capability than a crewed mission to the surface of Mars.

*Options for a crewed mars initiative*

- *Robotic and orbital/moon missions* This scenario involves a much larger array of robotic explorers being sent to Mars than NASA currently has. In addition, crewed missions would be sent to orbit Mars and possibly to Phobos and Deimos. While this option does not involve a crewed landing on the surface of Mars, the astronauts in orbit would be able to remotely operate robots on the surface in a much more efficient and directed manner than teams on Earth.

Since this is the least intensive option in terms of scale, it is also the least expensive and involves the smallest amount of risk. Without the need for human-rated landing and takeoff vehicles, the amount of research and engineering that would need to be undertaken is a fraction of a mission involving a crewed landing, lowering cost and making this scenario possible on a fairly short timescale.

The absence of setting humans on Mars also results in a substantial reduction in the risk to the astronauts in many respects. The amount of science that can be done pales in comparison to any mission with a crewed landing on the surface of Mars. This option also may be less exciting to the public than full human exploration missions.

- *Viking strategy* This scenario involves a small-scale crewed exploration mission that would set down on the surface of Mars and operate for several months before the crew would return to Earth. Eight astronauts would be selected to make the journey, and it would be launched at a time that would provide for not only a short travel time but also the shortest possible stay on the surface to minimize risk to the astronauts.

Having astronauts on the surface of Mars would greatly increase the relevance and amount of science data that the mission would yield compared to remote operation of robots. However, the technical and engineering hurdles that need to be addressed result in a major cost and timeframe increase. While risk would be minimized, it would still be substantial for all of the astronauts involved.

Without a permanent habitation plan, there is the risk that the mission will suffer a fate similar to the Apollo Program. That is, once we accomplish a crewed landing on Mars, interest and support in the Mars program may wane to the point of cancelling any future missions.

- *Pioneer strategy* This scenario involves a permanent settlement on the surface of Mars. This colony would be preceded by a fleet of robotic and supply ships that would deposit food, fuel, and materials on the surface. These robots would also begin preparations for constructing permanent habitats. An initial large crew of human explorers would be refreshed every few months both in terms of supplies and personnel.

A mission of this scale and duration would be able to unlock a large number of the mysteries we have concerning the history of Mars and the entire Solar System. Multiple locations could be settled or scouted, offering opportunities for an abundance of diverse scientific research. Humanity would become ‘Earth-Independent’, meaning that such a mission might no longer require support from our home planet and may become self-sustaining.

The technology and techniques required for such an undertaking would be extremely challenging. Methods of dealing with radiation, extracting water, producing fuel and air, propulsion, habitat construction, and a number of other techniques would need to be vastly improved before this scenario becomes feasible. It would involve a colossal increase over a smaller-scaled surface exploration mission in terms of cost, risk, and timeframe.

*The Asteroid Redirect Mission and the Proving Ground*

With the challenges for a crewed Mars mission defined and potential exploration scenarios outlined, it is obvious that the engineers and scientists of today need a framework that will allow for a successful Mars campaign. While some advocate laying an entire plan down from test missions through final exploration plans from the very beginning, there are other options for organizing the efforts that must be made. One of these options is the Proving Ground concept, which allows for missions to be designed, funded and carried out as NASA’s budget and capabilities dictate.

An example of the Proving Ground approach to Mars exploration was seen in the last discussion—NASA’s upcoming Asteroid Redirect Mission. While there is merit in the mission itself, it really serves primarily as a steppingstone towards the overarching goal of crewed Mars exploration. Many missions such as this would allow for the capabilities necessary for Mars exploration to be designed, tested, improved, and perfected on an increasing level of complexity and distance from the Earth. Smoothly transitioning from our current capabilities to the set of technologies and techniques necessary for a large-scale Mars initiative will likely be more cost-effective than planning an entire Mars mission start to end.

*Your Path to Mars Decision at the Forum*

During the Forum, you and other participants will discuss the different options for an eventual Mars exploration mission with a focus on the Proving Ground strategy. You will consider the options for a crewed Mars initiative in terms of scale, science benefits, cost, and even whether each option would be exciting or worthwhile. However, the main part of the discussion will focus on the more immediate future, and how the plan for Mars should be laid out as we move forward—would you like to see an entire strategy laid out now, or are you comfortable with a series of Proving Ground missions (such as the Asteroid Redirect Mission) that are undertaken as budgets and capabilities dictate?

### Questions Asked from Citizen Participants During the Forums

#### From the Phoenix Forum

- What exactly is the “extremely high cost” of a permanent colony on Mars?
  - ECAST
  - 35 min

It is too early to say exactly what the cost of a permanent settlement to Mars will be. What we do know is that permanent settlement will take longer to achieve than either Orbital/Robotic exploration or Viking boots on Mars and will cost more. In addition, NASA is working hard to reduce costs. One of the things are doing to lower the eventual cost of going to Mars is to focus on reusability of spacecraft and to develop the ability to use capabilities for when we go to Mars. NASA is looking for both commercial and international partners to help reduce the cost of any exploration mission.

- What is the likelihood that NASA will have the budget to accomplish these missions?
  - ECAST
  - 31 min
  - Clarification: NASA will use natural resources on Mars

It is never possible to fully predict future budgets, and NASA and others must do what they can today. NASA is striving to reduce costs by focusing on near term Proving Ground missions which draw off of the resources from multiple areas of NASA. For example, the ARM mission will draw off of the budgets in many different parts of NASA, including tech development, science, and human exploration. This, plus potential partnerships, will help make future exploration affordable.

- Why haven't we brought robots back from Mars?
  - ECAST
  - 27 min

It requires landing additional systems and propellant on Mars, which would be expensive and difficult to launch. We have not been able to do that to date, but NASA wants to do sample return from Mars, which would send back materials from the Mars surface, in the 2020s.

- Could we realistically bring humans back from Mars, or is it likely to be a one-way trip?
  - ECAST
  - 24 min

The challenges of bringing humans back from Mars are great due to the propellant, landing system and habitation challenges described above. But sending crews to Mars is realistic. Among its other activities to develop the ability to go to Mars, NASA's Mars 2020 payload will attempt to prove out ways to utilize Mars' natural resources to support future exploration.

NASA is online.

#### From Boston Forum

- How will they supply (under Pioneer strategy) every 3 months, when they can only come and go every 30 days or 500 days?
  - ECAST
  - Nov 15, 3:37 PM

You could resupply more frequently than every 500 days. There are optimal times to send payloads to Mars when the orbits of Earth and Mars are in alignment. This occurs roughly every two years. However, you can still send payloads more frequently than that if you use enough propellant and mass.

- What is the realistic difference between how much a robot can learn vs a human?
  - ECAST
  - Nov 15, 3:43 PM

A human is able to rapidly react to changing circumstances. Robots currently needs to be carefully designed to perform planned functions, sometimes autonomously. Having a person able to control a robot from close proximity (without the time delay to Mars) would allow for faster improvisation and could better enhance what research is done.

- What mysteries will be revealed? What will we glean from these initiatives?
  - ECAST
  - Nov 15, 3:58 PM

Mars once had an atmosphere similar to Earth but lost it. Why did this happen? It may have had life at one point—if we can find it, did it develop in similar ways to life on Earth? If we understand how life developed on Mars, we can understand if/how life may exist throughout the universe. There may also be new mysteries that we will discover when we arrive there. One can also comment about economic dimensions of humans expanding into the solar system. These initiatives could open up the solar system into the broader economic sphere. If humans can live off planet for significant periods, there may be other resource opportunities that humans can utilize.

## Footnotes

1 It should be clear that pTA can serve as a decision support tool to existing elected officials. A republican form of government, with representatives who are elected to represent their constituents, adds a layer of complexity to the discussion of how participatory discussions can inform decision making in a direct democracy. Elected representatives speak regularly with constituents, hold hearings, and can elicit citizen views in more direct ways. But they may still hear primarily from citizens who have a strong stake in issues being discussed. pTA can provide a way for representatives to learn from a broader sampling of the public about their goals.

2 Notable models that will not be discussed here are: Adam et al. (2006), Jassanoff (2012), Weisberg and Muldoon (2009) and Zollman (2007). The classic work of Kuhn (1962) and Merton (1973) give a more complete picture of the many science policy models available in the literature.

3 We thank an anonymous reviewer for pointing this out to us.

4 This idea is embraced in the work of Collins and Evans (2017), who take any prominent participation or endorsement of experts—as opposed to mere recognition—as a step towards a technocracy, preventing the embrace of a more thoughtful ‘elective modernism’ (Collins and Evans 2017, p. 71). For a more careful criticism of technocratic approaches, see Sclove (2012), who discusses things such as shared biases among experts and the need to incorporate public values in decisions.

5 In Kitcher (2011), there is a further distinction between ‘practical significance’ and ‘theoretical’ or ‘epistemic significance’. The former takes that the aims of science are about increasing anyone’s chance of attaining practical goals (Kitcher 2011, p. 64). The latter, instead, takes the aims of science to be those “that will mark out those truths the knowledge of which is intrinsically valuable” (Kitcher 2011, p. 65).

6 The aims of science here are considered on at least two levels, namely, the aims of science as a whole and the aims of a particular research project. For more on this, see Douglas (2014).

7 Kitcher takes curiosity as a balance between advancing human welfare—rather than knowledge—with mutual interest. Although hard to specify, curiosity seems to lack the format of random and reckless spirit of inquiry, as well as of a pure instrument for advancing science and technology (Kitcher 2001, p. 111).

8 It must be mentioned that Kitcher’s argument does not depend on abandoning the idea that science aims at true, objective knowledge of the world. He dedicates chapter 2 and 3 to flesh out the idea of a value-laden and epistemic sound scientific practice (Kitcher 2001).

9 Kitcher also rules out any genetic-based law. In this respect, he says: “Molecular genetics tells us at a staggering rate about the chemical structures of *individual* genes but fails to provide a general specification. Matters are even worse when we move away from genetics and consider such important biological notions as *cell*, *organism*, *Drosophila melanogaster*, *species*, and *predator*” (Kitcher 2001, p. 70, Emphasis in original).

10 See Kitcher (2001, p. 79) for a depiction of the significance graph for developmental biology with Dolly at its center.

11 This is of course not to say that the ideals of success are misguided, and thus scientific decisions are made based on moral, social, political, or religious values. For a more detailed discussion on values in science and the value-free ideal, see Douglas (2009).

12 For a controversial discussion on ideal deliberators, see Mouffe (2009) and van Bouwel (2015).

13 Harry Collins and Robert Evans think, erroneously to our mind, that citizens are tutored by experts (Collins and Evans 2017). The notion of expert is not entirely clear in Kitcher’s work. On this point, Kitcher suggests that an expert is someone to whom citizens, in the process of deliberating and being tutored, defer on scientific matters. But in any case, the relationship tutored-expert is not the one that Collins and Evans are pointing out. Instead experts do not play an absolute and defining role in WOS, but rather it is complementary to the process of tutoring. Mark Brown points this out by saying: “They [deliberators] then consult with experts to learn whether different lines of inquiry will actually realize the desired goals” (Brown 2004, p. 81). Brown’s ideas have been very influential to one of the authors (i.e., Pirtle).

14 More on issues on closure on debates, see Engelhardt and Caplan (1987).

15 We are leaving open the question of *who* decides what is scientifically significant for a pTA. There are several candidates, however. In our case, it could simply be the NASA directory, composed of engineers, directives, stakeholders, scientists, and other experts. As mentioned earlier, Kitcher suggests that questions of scientific significance do not necessarily need to have a practical or theoretical dimension. Many could be attached to fulfilling human curiosity (Kitcher 2011, p. 111). We believe that it is important to involve the public in assessing what values matter underneath scientific research, deepening and challenging presumptions about what is significant.

16 In this sense, the burden of evidence is on the critics of pTA who claim a need for participants to have sufficient expertise before they can deliberate on a topic. What, exactly, is lacking? If someone is tutored in Kitcher’s sense and has some basic historical sense of how a topic came to hold significance, then we think there is likely a fundamental rationale to allow that citizen to participate in such debates.

17 The extent to which real-world pTA efforts meet this could be a topic of its own study, but many studies of pTA do find that a collegial atmosphere can take hold. Observers placed in the NASA pTA example discussed below felt that this was the case for the eight tables with research observers.

18 The idealizations in WOS do not assume that each deliberant becomes an expert in an area, with access to unlimited information. The WOS thought experiment can only function if the deliberants themselves are able to judge if they are capable of making a decision.

19 WOS does have an important role for experts: after citizens have debated upon the goals that science should achieve, experts are brought in to decide on the means for how to pursue that. But, for our purposes of giving guidance to pTA efforts that focus on identifying values and goals that research projects should aspire to, then the WOS goal-setting component does not substantially rely on experts.

20 One of the authors, Pirtle, was one of the NASA collaborators on the project. Views and opinions expressed here are the authors’ and do not necessarily reflect the views of NASA or the United States Government.

21 For more, see Longino (1990). Donald Mackenzie’s book *Inventing Accuracy* also explores the complexity of social and technical influence (Mackenzie 1993).

22 We say this to emphasize that a diverse mix of discussions on values and facts is likely desirable for a future pTA, not as an effort to say that the NASA ECAST pTA was without flaw or ability to improve. The papers discussing the forum, referenced above, do note several ways in which the effort could be improved upon.

23 One issue that shall remain unanswered here is whether we should adopt Douglas’ strategy and demand that the scientists themselves take responsibility for value-judgments (Douglas 2003) or should we extend pTA to being more comprehensive and leaving more topics on the table for common citizens to discuss? We thank an anonymous reviewer from bringing this point to our attention.

24 This value map is not as rich as Kitcher’s (2001) significance graphs, identified above, but could eventually lead to that with the help of the historical efforts described later.

25 We can note that in a separate deliberation section, one discussion was sidelined as a result of a participant becoming confused by the material. It is possible for citizens to feel as though they do not have expertise. Given that the material used here was similar to material used by NASA managers, it’s expected that some reasonable level of information is being presented.

26 An anonymous reviewer did ask us whether some topics are potentially too divisive to be amenable to citizen deliberation (perhaps this might include issues of racism or climate change, especially if extreme positions on a given viewpoint are present). Forum facilitators do push for politeness and to make sure no one view dominates a table. However, divisiveness can likely become an issue in some pTA forums, just as some juries occasionally have hung trials due to strong disagreements among jurors. ECAST has done some deliberations on climate resilience, which may be a sign that more divisive topics can be handled here. We suspect that if a greater culture for citizen deliberation arises based on conversations in relatively non-controversial areas, it could help to create an ability to hold topics on the most divisive questions (some have quipped about pTA exercises as being akin to serving as ‘training wheels’ that may prepare citizens for potentially meatier democratic engagement).

27 Pirtle and Szajnfarber (2017) question the potential over-emphasis by scholars to try to create macro-ideals of how science and society relate and suggest focusing on more narrow ideals that can advise on specific instances. This paper follows that guidance by focusing primarily on a narrower epistemic standard for citizens involved in pTA, while not trying to reinvent a new marco-ideal.
